# The antibacterial efficiency of Chitosan photodynamically activated Phycocyanin, and Morinda Oleifera against S.mutans and the bonding strength between composite resin and caries-affected dentin

**DOI:** 10.12669/pjms.40.5.8800

**Published:** 2024

**Authors:** Amer M. Alanazi, Azmat Ali Khan, Amer Mahmood, Mohammad Amjad Kamal, Esha Abrar Baig

**Affiliations:** 1 Amer M. Alanazi, Pharmaceutical Biotechnology Laboratory, Department of Pharmaceutical Chemistry, College of Pharmacy, King Saud University, Riyadh 11451, Saudi Arabia; 2 Azmat Ali Khan, Pharmaceutical Biotechnology Laboratory, Department of Pharmaceutical Chemistry, College of Pharmacy, King Saud University, Riyadh 11451, Saudi Arabia; 3 Amer Mahmood, Stem Cell Unit Department of Anatomy, College of Pharmacy, King Saud University, Riyadh, Saudi Arabia; 4 Mohammad Amjad Kamal, Institutes for Systems Genetics, Frontiers Science Center for Disease-related Molecular Network, West China Hospital, Sichuan University, China and Enzymoics, 7 Peterlee Place, Hebersham, NSW 2770; Novel Global Community Educational Foundation, Australia; 5 Esha Abrar Baig, Department of Operative Dentistry, Dow University of Health Sciences, Karachi, Pakistan

**Keywords:** Phycocyanin, Chitosan, Moringa Oleifera, bond integrity, Photodynamic therapy

## Abstract

**Objective:**

Evaluation of contemporary disinfection techniques, Moringa Oleifera (M.Oleifera), Phycocyanin activated by photodynamic therapy (PDT), and Chitosan, on S.mutans survival rate and bond integrity of composite to carious-affected dentin (CAD).

**Methods:**

The in vitro study was conducted at King Saud University and concluded within three months. Sixty mandibular teeth with cavities extending to the middle third of the dentin were sterilized. *S.mutans* was inoculated onto the CAD surface of twenty samples. The samples were randomly divided into four groups (n: 15) based on various disinfection regimes. Group-1 received 2% CHX, Group-2 Phycocyanin activated by photodynamic therapy (PDT), Group-3 Chitosan, and Group-4 M.oleifera. S.mutans survival rate was calculated. Ten CAD samples from each group were restored using composite. The bond integrity of samples was assessed using a Universal testing machine (UTM) and failure mode using a stereomicroscope. Analysis of variance (ANOVA) and Tukey’s Post Hoc test were used to calculate statistical significance (p=0.05).

**Results:**

Group-2 samples subjected to Phycocyanin activated using PDT, displayed minimal survival rate (0.24 ± 0.05 CFU/ml) of S.mutans.Group-1 samples treated with CHX exhibited the highest count of S.mutans (0.69 ± 0.12 CFU/ml). The most robust bond was observed in Group-3 (Chitosan) samples (19.33 ± 0.47 MPa). In contrast, SBS values were lowest in Group-1 (CHX) treated study samples (13.17 ± 1.88 MPa).

**Conclusion:**

Chitosan, Phycocyanin activated by PDT, and Moringa Oleifera extract exhibit potential as viable substitutes for chlorhexidine (CHX) in clinical settings, presenting the possibility of better eradication of S.mutans and greater adhesive strength to CAD.

## INTRODUCTION

Streptococcus mutans (S. mutans) is a prevalent bacterium in the oral cavity that plays a substantial role in the development of dental caries. *S. mutans* exhibits exceptional proficiency in the metabolism of carbohydrates, resulting in the production of acid as a secondary product. The acid corrodes the outer layer of the teeth, causing the loss of minerals and ultimately resulting in the formation of cavities.^1^Cr:YSGG lased dentin in comparison with other conditioning regimes. Materials and methods: One hundred twenty extracted teeth were mounted and allocated into eight groups (n = 15 The choice to employ the CAD surface for adhering restorative material has posed difficulties, resulting in a decrease in bonding ability and affecting the overall longevity and effectiveness of the restorations.^2^Sodium hypochlorite (NaOCl To deal with this issue, it has been proposed to implement a CAD disinfection routine before the application of restorative material.^3^

Chlorhexidine (CHX) is widely recognized as an extremely efficient antibacterial disinfectant and is commonly referred to as the “gold standard.”^4^ Based on the data that is currently available, it has been discovered that it can inhibit matrix metalloproteinase (MMP), which helps to maintain the long-term stability of the restoration.^5^,^6^ Moreover, other frequent issues include alterations in taste perception and dentin discoloration. CHX, known for its bitter taste, may result in temporary changes in taste perception for patients after application.^5^,^6^

Photodynamic therapy (PDT) in restorative dentistry is being explored as an adjunctive or alternative treatment for conditions such as dental caries, periodontal diseases, and other oral infections.^7^ It is considered a minimally invasive and targeted approach, with the potential to enhance the outcomes of conventional dental treatments.^8^ Recently, researchers have shown interest in a novel PS called Phycocyanin (PC), a light-harvesting pigment found in *Spirulina Platensis*, due to its intrinsic anti-inflammatory, anti-cancer, and antioxidant properties.^9^ Similarly, another CAD cleaner, called ‘Chitosan,’ a polysaccharide-based substance made up of copolymers of glucosamine and N-acetylglucosamine. Chitosan is a strong chelating agent that is created by partially deacetylating chitin.^10^ It has been successfully employed to remove decayed areas by restoring their mineral content through the use of calcium and phosphate.^11^ Furthermore, it has the potential to initiate the production of crystals on the dentin surface. Nevertheless, there is a lack of evidence regarding their influence on the bond values of resin adhesive and antibacterial effectiveness against S.mutans, necessitating more inquiry.

Moringa Oleifera (M. Oleifera) is recognized for its elevated levels of β-carotene, a precursor to vitamin A and a potent antioxidant that safeguards cells from free radical-induced damage. The leaf extract of M.Oleifera is abundant in β-carotene, vitamin E, and protein, establishing it as a viable source of antioxidants.^12^ Numerous studies have extensively detailed the anti-inflammatory, antibacterial, and anticancer properties found in the leaf extract.^13^,^14^ However, there is a lack of literature exploring the impact of S.mutans survival rate and SBS of composite restoration bonded to CAD.

The objective of the present study was to explore a notable disparity in the survival rates of S.mutans on CAD after implementing contemporary disinfection techniques, including Chitosan, Phycocyanin activated by PDT, and M.Oleifera leaf extract, in comparison to CHX. It was anticipated that specimens treated with Chitosan, PDT activated Phycocyanin, and M.Oleifera extract would demonstrate similar bond strength between composite and CAD when compared to samples treated with CHX. Hence, the primary aim of this modern investigation was to examine the influence of the most recent disinfection methods on the rates of survival of S.mutans and SBS to CAD.

## METHODS

The in-vitro study, carried out at King Saud University, concluded within three months. The leaves of *M.oleifera* underwent air drying at room temperature until a constant weight was achieved. Following desiccation, the dried leaves were finely powdered and subjected to extraction using a soxhlet apparatus. The extraction process involved utilizing ten volumes of 80% (v/v) methanol in batches of 50 grams, resulting in the formation of the methanolic extract. Subsequently, the extract was evaporated to eliminate the solvent under reduced pressure at a temperature of 40°C through the use of a rotary evaporator. The raw methanolic extract was then mixed with 100 ml of distilled water and fractionated into ethyl acetate (3 x 100 ml). The ethyl acetate fraction obtained was evaporated to dryness under vacuum at 40°C using a rotary evaporator and further subjected to freeze-drying. A 0.22 m pore-size polyvinylidene fluoride (PVDF) filter was employed to filter the extracts. The filtered extract was refrigerated at a temperature of 4°C for future utilization.

Among sixty samples, twenty CAD surfaces were deliberately exposed to S.mutans, which were used as the microorganisms being tested in the study. This goal involved the utilization of clinical isolates of S.mutans. A solitary colony of S.mutans was added to a 5ml solution of brain heart infusion broth (BHI-broth) in a vial with a screw cap. Subsequently, all the vials were placed in an incubator set at a temperature of 37°C for one day. A 0.5 ml bacterial solution was introduced into a vial with a screw lid, which already contained 0.5 ml of BHI-broth. This led to a final concentration of 4×107 CFU/ml. Before inoculation, the CAD surface was subjected to drying, followed by the application of a bacterial suspension containing 4×105 CFU, with a volume of 10 μl, onto the surface. The contaminated specimens were subjected to incubation at a temperature of 37°C for 24 hours. After receiving the disinfection regimen, the study samples were randomly assigned to one of four groups, with each category including 15 samples (n=15 for each group).

*Group-1 (Control):* In this group, carious-affected dentin (CAD) samples underwent treatment with a 2% chlorhexidine (CHX) solution (Varni Corporation, Gujrat, India) for 60 seconds. Subsequently, the disinfected surface was cleaned using distilled water and air-dried for five seconds. *Group-2:* The sample was subjected to a treatment with 100 µL of chitosan solution at a concentration of 3 mg/ml for 60 seconds. Afterward, the CAD surface was washed with distilled water and dried using air for five seconds. *Group-3:* A new stock solution was created by dissolving PC powder (Photoactive+, Weber Medical, Germany) in distilled water. The resultant mixture was kept in a lightless setting at ambient temperature until it was needed. The Phycocyanin was activated by employing a 635-nm diode laser (Konftec, Taiwan) in continuous mode for three minutes. The laser had a power output of 220 mW and a power density of 0.34 W/cm².^15^ *Group-4: In* this group, the CAD surface underwent treatment with Moringa Oleifera leaf extract for one minute, ensuring that no desiccation occurred. Subsequently, the surfaces were dried using compressed air from a triple syringe.^13^,^16^

*Survival rates =* Colony forming units (CFUs) of each experiment group/ CFU count of the positive control

### Bonding procedure

The disinfected samples from all groups were bonded with Composite resin, (A2 shade of Z350XT 3M ESPE in St. Paul, MN, USA) compacted into the tube using a plugger. The composite restoration was applied incrementally at 2mm thickness and cured for 20 seconds delivering a light intensity (Demi Plus; Kerr, USA) of 1100 mW/cm².

### Assessment of SBS and failure mode

The specimens were placed onto a Universal Testing Machine (UTM). Stress was applied at the junction between the teeth and the restorative material utilizing a knife-edge blade, subjecting them to a 2 kN load at a crosshead speed of 1-mm per second. The force required to debond the restoration was measured in Megapascals (MPa). Subsequently, the failure mode was assessed using a stereomicroscope at a magnification of 40x^1^Cr:YSGG lased dentin in comparison with other conditioning regimes. Materials and methods: One hundred twenty extracted teeth were mounted and allocated into eight groups (n = 15

### Ethical approval

The study was approved by the Institutional Review Board with the reference IRB# F9-948X8 on June 3, 2023.

### Statistical analysis

SPSS 18.0.0 software (SPSS Inc.Chicago, USA) was used for statistical analysis. Analysis of variance (ANOVA) and Tukey’s test, with a significance level of p=0.05, were used to assess statistical significance.

## RESULTS

**Fig.1:**
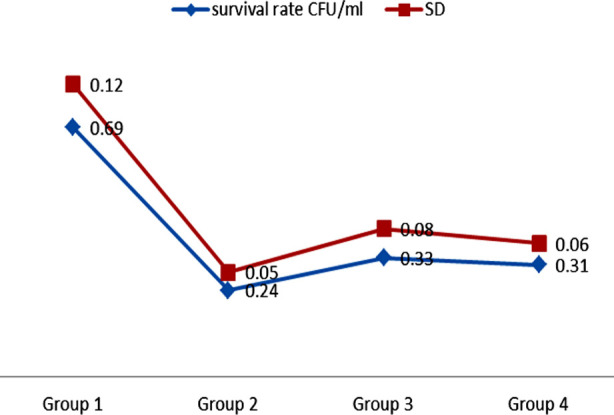
Mutans survival rates after different methods of disinfection

The findings demonstrated that Group-2 samples, which were subjected to Phycocyanin activated using PDT, displayed the most minimal survival rate (0.24 ± 0.05 CFU/ml) of S.mutans. In contrast, the Group-1 samples that were pre-treated with CHX exhibited the highest count of surviving microorganisms (0.69 ± 0.12 CFU/ml). Intergroup comparison analysis showed that Group-2 (Phycocyanin activated by PDT), Group-3 (Chitosan) (0.33 ± 0.08 CFU/ml), and Group-4 (M. Oleifera) (0.31 ± 0.06 CFU/ml) did not exhibit any significant variation in their effectiveness as antimicrobial agents against S.mutans (p>0.05). Group-1 demonstrated considerably reduced levels of S.mutans survivability in comparison to other modern disinfection treatments (Fig.1).

**Fig.2:**
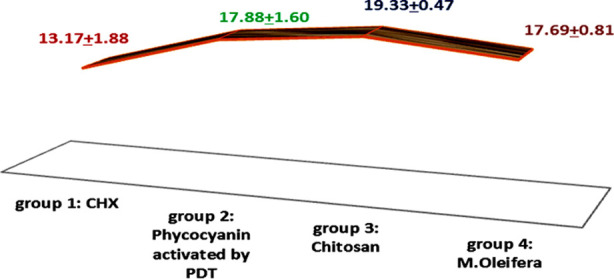
Means and standard deviations (SD) for bond strength values among study groups

**Fig.3:**
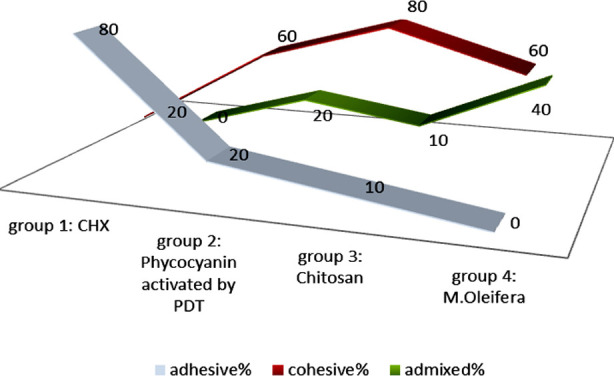
Modes of failure among study groups

The results of the bond strength at the composite-CAD interface following the application of different disinfecting agents revealed that the most robust bond was observed in Group-3 (Chitosan) samples (19.33 ± 0.47 MPa). In contrast, the shear bond strength (SBS) values were lowest in Group-1 (CHX) treated study samples (13.17 ± 1.88 MPa). Intergroup comparison analysis demonstrated that Group-2 CAD disinfected with Phycocyanin activated by photodynamic therapy (PDT) (17.88 ± 1.60 MPa), Group-3 (Chitosan) (19.33 ± 0.47 MPa), and Group-4 (M.oleifera) (17.69 ± 0.81 MPa) exhibited no significant difference in their attained bond integrity values (p > 0.05)Fig.2. The failure mode among different experimental groups is shown in Fig-3. Group-1 showcased predominantly adhesive failure type.Group-2, Group-3, and Group-4 specimens mainly demonstrated cohesive failure.

## DISCUSSION

The laboratory-based investigation aimed to assess whether there was a significant difference in the survival rates of S.mutans on the carious-affected dentin (CAD) surface when utilizing contemporary disinfection strategies, including Chitosan, Phycocyanin activated by photodynamic therapy (PDT), and M.oleifera extract, compared to the traditional control method CHX. Additionally, the study expected comparable shear bond strength scores between composite and CAD for specimens treated with Chitosan, Phycocyanin activated by PDT, and M.oleifera leaf extract compared to those treated with CHX. The hypothesis was fully rejected, as Chitosan, Phycocyanin activated by PDT, and M.oleifera disinfected specimens exhibited superior antibacterial activity and bond strength values compared to CHX.

Regarding survival rates of *S.Mutans*, it was observed that Chitosan, *M.Oleifera, and* Phycocyanin activated by PDT presented a minimum count of *S.Mutans* surviving on the CAD surface. Chitosan operates by modifying the bacterial cell wall and cell membrane permeability, impeding S.mutans from adhering to tooth surfaces. This prevents the multiplication and emergence of S.mutans, ultimately inhibiting biofilm formation.^17^,^18^ Moreover, The ethanol-based leaf extract derived from M.Oleifera has shown notable potential in effectively eliminating S.mutans. This promising antimicrobial effect can be attributed to the diverse array of constituents present in the M.Oleifera leaf extract, which includes proteins, fatty acids, and phenolic compounds. The observed efficacy against S.mutans is likely a result of the combined action of these bioactive components. Proteins play a role in disrupting microbial structures, fatty acids exhibit antimicrobial properties by affecting cell membranes, and phenolic compounds are known for their antioxidant and antibacterial activities. The synergistic interaction of these constituents contributes to the overall antimicrobial potency of the ethanol-based M.Oleifera leaf extract.^14^,^16^ Likewise, PDT-activated Phycocyanin can induce disruption of the cell membrane and DNA cleavage in microorganisms, causing disturbances in their cell energy metabolism resulting in lysis of S.mutans. Following activation of Phycocyanin, there is a notable increase in ROS production, enhancing its antimicrobial potential.^9^,^15^ On the contrary, the current study revealed a higher survival rate of S.mutans following CHX disinfection. Plausible explanations for this outcome include incomplete disinfection, the potential formation of persister cells, and bacterial adaptation over time. These factors suggest that the disinfection process might not have been fully effective, allowing for the persistence of S.mutans in the treated CAD surface.^19^

Achieving satisfactory bond strength scores is crucial for dental adhesion, and this requires careful attention to the adhesive’s interaction with the dentin substrate. It is imperative that the applied adhesive thoroughly permeates the collagen matrix of dentin homogeneously and undergoes efficient polymerization.^20^ Favourable bond scores were noted after CAD disinfection using a Phycocyanin activated by PDT. A plausible explanation for this phenomenon was provided by an in vitro trial conducted by Hashemikamangar and colleagues.^15^ They proposed that one potential factor influencing the results could be the lower and biocompatible level of reactive oxygen species (ROS) produced by the photosensitizer (PS) irradiation.^15^ Similarly, samples treated with M.oleifera also demonstrated a favorable effect on bond integrity outcomes. In a laboratory-based study conducted by Mona Shaaban and Nawal Aidaros, it was observed that the bond strength between composite materials and bleached enamel was not only higher but also clinically satisfactory after pretreatment with M.Oleifera as an antioxidant. This finding suggests that the application of M. Oleifera as a pretreatment contributed positively to the adhesion between dental composite and tooth structure. The use of antioxidants, such as M.Oleifera, in dental applications is notable for their potential to mitigate the adverse effects of oxidative stress induced by bleaching agents. Oxidative stress can compromise the tooth structure and affect the subsequent bond strength between restorative material and CAD.^21^

The enhanced bond integrity observed following CAD disinfection with chitosan can be attributed to a study conducted by Ramasetty and colleagues.^22^ Their findings suggested that chitosan when used as a cavity disinfectant, enhances the sealing ability and promotes the formation of resin tags between the composite and dentin.^22^ These results align with the outcomes reported in the study conducted by Porenczuk et al.^23^ Furthermore, the lower bond integrity observed with CHX may be explained by its potential to obstruct dentinal tubules, possibly due to debris on the carious-affected dentin (CAD) structure.^5^

Upon evaluating the failure modes, it was found that the samples in Group-1 primarily displayed an adhesive kind of failure. Specimens from Group-2, Group-3, and Group-4, on the other hand, predominantly exhibited coherent kinds of failure. Cohesive failure can be ascribed to multiple sources, such as material deficiencies, brittleness, excessive load, elevated stresses, chemical deterioration, material aging, and wear.^2^,^24^,^25^ These data indicate that the disinfection procedures employed in Group-2, Group-3, and Group-4 may enhance the bonding of materials at the interface of the composite and carious impacted dentin.

### Limitations

The current study has certain limitations, notably the absence of existing literature examining the changes in dentin surfaces after the application of Chitosan, Phycocyanin activated by PDT, and M.oleifera using advanced imaging techniques such as scanning electron microscopy (SEM) and atomic force microscopy (AFM). Addressing these gaps in the literature through future research studies is crucial to gaining a more comprehensive understanding of the effects of these materials on dental health.

## CONCLUSION

Chitosan, Phycocyanin activated by PDT, and Moringa.Oleifera extract exhibits potential as a viable substitute for chlorhexidine (CHX) in clinical settings, presenting the possibility of better eradication of S.mutans and greater adhesive strength. Nevertheless, it is imperative to underscore the necessity for additional comprehensive research and investigations to definitively validate these advantages.

### Authors` Contribution:

**AM, AMA, AAK, and EAB: S**tudy design, Data collection and interpretation manuscript writing, final manuscript approval. **AM, AMA, EAB:** Data analysis, Writing and Revision.All authors are responsible and accountable for the accuracy and integrity of the work.

## References

[1] Alkhudhairy F, Vohra F, Naseem M (2020). Influence of Er,Cr:YSGG Laser Dentin Conditioning on the Bond Strength of Bioactive and Conventional Bulk-Fill Dental Restorative Material. Photobiomodulation Photomedicine Laser Surg.

[2] Abrar E, Naseem M, Baig QA, Vohra F, Maawadh AM, Almohareb T (2020). Antimicrobial efficacy of silver diamine fluoride in comparison to photodynamic therapy and chlorhexidine on canal disinfection and bond strength to radicular dentin. Photodiagnosis Photodyn Ther.

[3] Nisar SS, Irfan F, Hammad H, Abdulla AM, Kamran MA, Barakat A (2022). Disinfection of caries-affected dentin using potassium titanyl phosphate laser, Rose bengal and Ozonated water on shear bond strength of deciduous teeth. Photodiagnosis Photodyn Ther.

[4] Borges FM, De Melo MA, Lima JP, Zanin IC, Rodrigues LK (2012). Antimicrobial effect of chlorhexidine digluconate in dentin:In vitro and in situ study. In:Journal of Conservative Dentistry Vol 15. Wolters Kluwer -- Medknow Publications.

[5] Aykut-Yetkiner A, Candan U, Ersin N, Eronat C, Belli S, Özcan M (2015). Effect of 2% chlorhexidine gluconate cavity disinfectant on microtensile bond strength of tooth-coloured restorative materials to sound and caries-Affected dentin. J Adhes Sci Technol.

[6] Al Deeb L, Bin-Shuwaish MS, Abrar E, Naseem M, Al-Hamdan RS, Maawadh AM (2020). Efficacy of chlorhexidine, Er Cr YSGG laser and photodynamic therapy on the adhesive bond integrity of caries affected dentin. An in-vitro study. Photodiagnosis Photodyn Ther.

[7] Alkhudhairy F, Naseem M, Ahmad ZH, Alnooh AN, Vohra F (2019). Influence of photobio-modulation with an Er,Cr:YSGG laser on dentin adhesion bonded with bioactive and resin-modified glass ionomer cement. J Appl Biomater Funct Mater.

[8] Almadi KH, Alkahtany MF, Almutairi B (2021). Influence of synthetic and natural photosensitizers activated by photodynamic therapy on extrusion bond strength of fiber post to radicular dentin. Pak J Med Sci.

[9] Chiniforush N, Pourhajibagher M, Parker S, Benedicenti S, Bahador A, Salagean T (2020). The effect of antimicrobial photodynamic therapy using chlorophyllin-phycocyanin mixture on enterococcus faecalis:The influence of different light sources. Appl Sci.

[10] Kim DA, Lee JH, Jun SK, Kim HW, Eltohamy M, Lee HH (2017). Sol–gel-derived bioactive glass nanoparticle-incorporated glass ionomer cement with or without chitosan for enhanced mechanical and biomineralization properties. Dent Mater.

[11] Wieckiewicz M, Boening K, Grychowska N, Paradowska-Stolarz A (2016). Clinical Application of Chitosan in Dental Specialities. Mini-Reviews Med Chem.

[12] Khallaf ME, Kataia EM, Aly Y, Omar N, Mohamed MA (2020). Cleanliness efficacy and effect on dentin microhardness of a novel plant extract irrigant. Bull Natl Res Cent.

[13] Duarte K, Thomas B, Varma SR, Kamath V, Shetty B, Kuduruthullah S (2022). Antiplaque Efficacy of a Novel Moringa oleifera Dentifrice:A Randomized Clinical Crossover Study. Eur J Dent.

[14] Segwatibe MK, Cosa S, Bassey K (2023). Antioxidant and Antimicrobial Evaluations of Moringa oleifera Lam Leaves Extract and Isolated Compounds. Molecules.

[15] Hashemikamangar SS, Alsaedi RJF, Chiniforush N, Motevaselian F (2022). Effect of antimicrobial photodynamic therapy with different photosensitizers and adhesion protocol on the bond strength of resin composite to sound dentin. Clin Oral Investig.

[16] Jwa SK (2019). Efficacy of moringa oleifera leaf extracts against cariogenic biofilm. Prev Nutr Food Sci.

[17] Parolia A, Kumar H, Ramamurthy S, Davamani F, Pau A (2020). Effectiveness of chitosan-propolis nanoparticle against Enterococcus faecalis biofilms in the root canal. BMC Oral Health.

[18] Ong TH, Chitra E, Ramamurthy S, Siddalingam RP, Yuen KH, Ambu SP (2017). Chitosan-propolis nanoparticle formulation demonstrates anti-bacterial activity against Enterococcus faecalis biofilms. PLoS One.

[19] Borges FM, De Melo MA, Lima JP, Zanin IC, Rodrigues LK (2012). Antimicrobial effect of chlorhexidine digluconate in dentin:In vitro and in situ study. J Conserv Dent.

[20] Alshahrani A, Abrar E, Maawadh AM, Al-Hamdan RS, Almohareb T, AlFawaz Y (2020). Management of caries affected dentin (CAD) with resin modified glass ionomer cement (RMGIC) in the presence of different caries disinfectants and photosensitizers. Photodiagnosis Photodyn Ther.

[21] Mohamed M, Aidaros N (2020). Assessment the impact of moringa oleifera extracts and ascorbic acid solution as antioxidants on microshear bond strength of resin composite to bleached bovine enamel (In vitro study). Egypt Dent J.

[22] Ramasetty PA, Tripathi AP, Sugandhan S, Naik SV, Deepak BM (2018). Nanotechnology in Dentin Disinfection:Can We Preserve the Bond?. Int J Clin Pediatr Dent.

[23] Porenczuk A, Grzeczkowicz A, Maciejewska I, Golas M, Piskorska K, Kolenda A (2019). An initial evaluation of cytotoxicity, genotoxicity and antibacterial effectiveness of a disinfection liquid containing silver nanoparticles alone and combined with a glass-ionomer cement and dentin bonding systems. Adv Clin Exp Med.

[24] Alkhudhairy F, Vohra F, Naseem M, Owais MM, Amer AHB, Almutairi KB (2020). Color stability and degree of conversion of a novel dibenzoyl germanium derivative containing photo-polymerized resin luting cement. J Appl Biomater Funct Mater.

[25] Alkhudhairy F, Vohra F, Naseem M, Ahmad ZH (2019). Adhesive bond integrity of dentin conditioned by photobiomodulation and bonded to bioactive restorative material. Photodiagnosis Photodyn Ther.

